# Correlation between Electronic Defect States Distribution and Device Performance of Perovskite Solar Cells

**DOI:** 10.1002/advs.201700183

**Published:** 2017-07-06

**Authors:** Giovanni Landi, Heinz Christoph Neitzert, Carlo Barone, Costantino Mauro, Felix Lang, Steve Albrecht, Bernd Rech, Sergio Pagano

**Affiliations:** ^1^ Dipartimento di Ingegneria Industriale (DIIn) Università di Salerno Via Giovanni Paolo II 132 84084 Fisciano (SA) Italy; ^2^ Dipartimento di Fisica “E.R. Caianiello” and CNR‐SPIN Salerno Università di Salerno Via Giovanni Paolo II 132 84084 Fisciano (SA) Italy; ^3^ Helmholtz‐Zentrum Berlin für Materialien und Energie GmbH Institut für Silizium Photovoltaik Kekuléstr. 5 12489 Berlin Germany

**Keywords:** electron–hole recombination, electronic disorder, electron–phonon interaction, noise spectroscopy, perovskite solar cells

## Abstract

In the present study, random current fluctuations measured at different temperatures and for different illumination levels are used to understand the charge carrier kinetics in methylammonium lead iodide CH_3_NH_3_PbI_3_‐based perovskite solar cells. A model, combining trapping/detrapping, recombination mechanisms, and electron–phonon scattering, is formulated evidencing how the presence of shallow and deeper band tail states influences the solar cell recombination losses. At low temperatures, the observed cascade capture process indicates that the trapping of the charge carriers by shallow defects is phonon assisted directly followed by their recombination. By increasing the temperature, a phase modification of the CH_3_NH_3_PbI_3_ absorber layer occurs and for temperatures above the phase transition at about 160 K the capture of the charge carrier takes place in two steps. The electron is first captured by a shallow defect and then it can be either emitted or thermalize down to a deeper band tail state and recombines subsequently. This result reveals that in perovskite solar cells the recombination kinetics is strongly influenced by the electron–phonon interactions. A clear correlation between the morphological structure of the perovskite grains, the energy disorder of the defect states, and the device performance is demonstrated.

## 1 Introduction

Perovskite materials have attracted considerable interests for solar cell applications due to their efficient and balanced ambipolar transport property and strong optical absorption coefficient.^1^ Amongst these compounds, the hybrid organic–inorganic perovskite methylammonium lead triiodide (CH_3_NH_3_PbI_3_) is the most widely studied composition.^2^ The possibility to fabricate a good polycrystalline thin film layer as light absorber, with smaller bulk crystal defects compared to the organic semiconductors, has produced an enhancement of the power conversion efficiency η of the thin film perovskite solar cell. The efficiency has increased from 3.8% to exceed 20% in less than 7 years, and with possible application in tandem devices.^3^, ^4^, ^5^ Recently, an efficient monolithic tandem cell formed by a silicon heterojunction bottom‐ and a perovskite top‐cell has been fabricated with low‐temperature processes and shows η ≃ 18%.^6^ In a mechanical stack perovskite‐silicon tandem, a 26.4% efficiency has been observed,^7^ which is about the same value as the most recent single junction solar cell record, with silicon as absorber material.^8^ In addition, these organic–inorganic perovskites exhibit radiation hardness and withstand proton doses that exceed the damage threshold of crystalline silicon by almost three orders of magnitude.^9^ The perovskite material exhibits two distinct phase transitions, one is located at low temperatures around 160 K (from orthorhombic to tetragonal phase) and the other one occurs at around 310–330 K when the tetragonal structure changes to cubic crystalline structure.^10^ Several authors report the influence of phase transitions on the performance of the perovskite solar cell.^11^, ^12^ Leong et al. have reported a monotonic increase of the power conversion efficiency as a function of the temperature up to 330 K where η decreases due to increased recombination.^11^ Additionally, Peng et al. have observed a rapid change of both hole and electron mobilities during phase transition from tetragonal to cubic crystalline structures at around 310–330 K until reaching a balance of hole and electron transport, which gives rise to the best cell performance.^12^

However, in contrast with the interesting optoelectronic properties of the CH_3_NH_3_PbI_3_ material, the understanding of the defect structure and their impact on carrier recombination and charge transport combined with the electron–phonon scattering is still an open issue.^13^, ^14^, ^15^ The presence of the intrinsic defects, especially point defects, in the absorber layer can lead to the formation of distributed recombination centers within the forbidden gap that could cause recombination losses. Additionally, the intrinsic point defects can contribute to the formation of shallow defects that produce an unintentional doping in the absorber layer.^10^

In literature several approaches have been proposed in order to mitigate the effect of the recombination process, through defect states, on the device performances.^3^, ^16^ In this respect, Chen et al. have reported that the use of chlorine as extrinsic dopants, during the solution crystallization and grain growth of CH_3_NH_3_PbI_3_, produces an enhancement of the film quality which results in improved efficiency.^17^ Moreover, Shao et al. have demonstrated that the use of the fullerene material, as electron transport layer, leads to a partial deactivation of the surface and of the grain boundary charge traps, related to the perovskite absorber layer. This also helps to eliminate the often observed photocurrent hysteresis and to increase the charge carrier lifetime and mobility.^3^ Several experimental techniques, such as temperature dependent admittance spectroscopy or time‐resolved photoluminescence decay measurements, have been used to obtain a detailed analysis of the defect states population and recombination processes.^10^, ^18^ Recently, low‐frequency noise spectroscopy has been applied to characterize charge transport in polymer/carbon nanotubes composites, thermal aging and degradation effects in bulk heterojunction polymer solar cells, and proton induced defect formation in Si‐based devices.^19^, ^20^, ^21^ More recently, the analysis of the temperature dependence of the noise amplitude has been also used to unravel the low‐temperature metastable state in perovskite solar cells.^22^

In this study, the charge carrier dynamics in the hybrid organic–inorganic perovskite solar cell has been evaluated under variation of temperature, bias current, and illumination level by using noise spectral density measurements. Current fluctuations, induced by the capture and emission of the charge carriers from the defect states, can be modeled by means of trapping/detrapping and recombination mechanisms. The effect induced by the room‐temperature tetragonal and the low‐temperature orthorhombic phase structures of the CH_3_NH_3_PbI_3_ material, and the nature of the charge trapping processes combined with the shallow and deep defect states have been considered. Additionally, the correlation between the grain size of the thin perovskite layer, the charge carrier transport in presence of a broad distribution of defect states, and their implications on the power conversion efficiency have been investigated in detail.

## 2 Results and Discussion

### 2.1 DC Electrical Characterization

**Figure 1:**
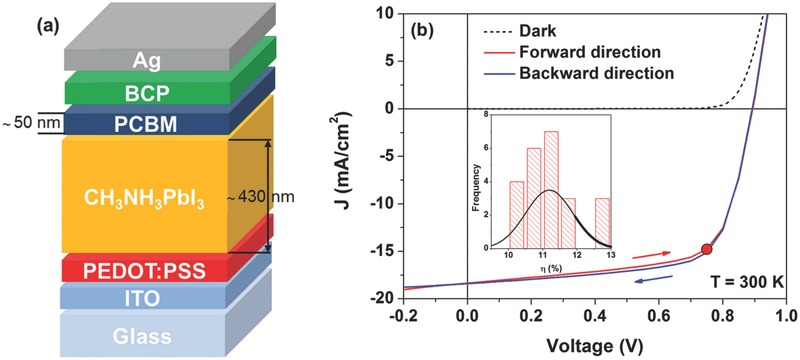
a) Schematic illustration of the cross‐section of the device structure. b) Current density–voltage characteristics measured at 300 K under dark conditions (dashed line) and under illumination (solid lines) showing no hysteresis for the perovskite solar cell. Red and blue lines refer to performed forward and backward voltage scans, respectively. The result of a maximum power point tracking after stabilization verifies the power conversion efficiency of 11.4% (red dot). The inset shows a histogram of the measured efficiency of 23 processed devices.

The perovskite solar cell is composed of a CH_3_NH_3_PbI_3_ absorber layer sandwiched between the electron and hole transport layers. **Figure** **1**a depicts the investigated device structure, following the layer sequence glass/ITO/PEDOT:PSS/CH_3_NH_3_PbI_3_/PCBM/BCP/Ag. Hereby ITO is indium tin oxide, PEDOT:PSS is poly(3,4‐ethylenedioxythiophene) polystyrene sulfonate, PCBM is [6,6]‐phenyl‐C61‐butyric acid methyl ester, and BCP is bathocuproine. The current density–voltage characteristics (*J*–*V*) of the perovskite solar cell measured at room temperature under dark conditions (dashed line) and under one sun illumination (solid lines) are shown in Figure 1b. The investigated device is characterized by the following parameters: short circuit current *J*_SC_ = (18.4 ± 0.5) mA cm^−2^, open circuit voltage *V*_OC_ = (892 ± 4) mV, fill factor FF = (69 ± 2)%, and power conversion efficiency η = (11.4 ± 0.5)%. The power conversion efficiency is further validated by maximum power point (MPP) tracking with stable values over 300 s, indicated as red dot. As can be observed in Figure 1b, the illuminated *J*–*V* curves exhibit a low value of photocurrent hysteresis, when measuring the characteristics in forward and backward voltage directions. This finding, already reported in literature, points out the usefulness of the PCBM material as efficient passivation layer for the large density of charge traps in the CH_3_NH_3_PbI_3_ material.^3^

Typically, the perovskite‐based solar cells are designed with an intrinsic absorber layer forming a p–i–n (or n–i–p) device architecture. Here, i stands for intrinsic and corresponds to the CH_3_NH_3_PbI_3_ material, whereas n and p corresponds to the electron and hole transport layers (i.e., PCBM and PEDOT:PSS, respectively).^10^ Several studies on the inverted perovskite solar cells (p–i–n) indicate the presence of a p–n heterojunction generated by electrical defects at the cathode, acting as p‐type dopants within the absorber layer.^23^, ^24^ The value of the built‐in voltage *V*_bi_, which takes into account the bandgap discontinuity at the cathode interface, can be estimated by considering the linear part of the *J*–*V* curve at *J* = 0. As shown in Figure S1 (Supporting Information), the investigated solar cells are characterized by a *V*_bi_ value of about 0.86 V at 300 K. For a similar device structure based on the junction between CH_3_NH_3_PbI_3_ and PCBM, values of *V*_bi_ ≈ 1 V at room temperature by using Mott–Schottky analysis have been reported.^23^, ^24^ This latter value will be used for the subsequent analysis. The differences in *V*_bi_ can be related to the formation of the dipole layer at the interface between the absorber material and the PCBM layer.^24^ The presence of defect states within the device strongly influences the recombination processes and, therefore, the dark current values.^25^

By fitting the *J*–*V* curves of the perovskite solar cell shown in Figure S2 (Supporting Information), a shunt resistance *R*_sh_ = 6.5 kΩ cm^2^, a series resistance *R*_s_ = 5 Ω cm^2^, and a diode ideality factor *n* = 1.5 can be estimated. As reported in literature, a value of *n* > 1 suggests that the perovskite dark current is dominated by carrier recombination. In particular, a part of the recombination processes are trap‐assisted by deep states related to the defects/impurities in the crystal bulk and/or along the grain boundaries.^4^, ^26^ Their occupation probability is then governed by the Shockley–Read–Hall (SRH) statistics.^27^

### 2.2 AC Voltage‐Noise Characterization

At low frequencies and under charge carrier injection, the ac equivalent electric circuit of the solar cell is simply composed by a parallel connection between the differential resistance $R_{D}\;=\;{\mathrm{dV}}_{F}\mathrm{/dJ}$ and the chemical capacitance *C*_μ_.^25^ In more detail, $V_{F}\;=\;V\;-\;R_{s}J$ is the forward bias voltage corresponding to the splitting of the quasi‐Fermi levels for electrons and holes (*E*_Fn_ and *E*_Fp_, respectively) under current biasing or light illumination. *C*_μ_ is a capacitance containing the contribution of the minority charge carriers stored in the device. In this framework, the effective lifetime τ_eff_ is expressed as the product *R*_D_ + *C*_μ_ and can be estimated by the noise frequency dependence, taking into account the dominant trap‐assisted recombination phenomena in the device.^25^ The noise spectroscopy provides an ac signal with a wide frequency bandwidth, where the observed fluctuations contain information about the material properties and the underlying charge carrier kinetics. The trapping of the charge carrier in the defect states can lead to a nonradiative recombination process or to an emission of the charge in the conduction band. The trapping/detrapping processes cause a number fluctuation of the charge carriers and, therefore, a current‐noise contribution that can be measured at the external contacts.^21^ In terms of spectral density, the resulting current‐noise source can be expressed as $S_{I}(f)\;=\;S_{V}(f){\mathrm{/R}}_{D}^{2}$, being *S*_V_ the measured voltage‐spectral density.^28^ As already reported for polymer:fullerene^25^ and Si‐based photovoltaic devices,^21^ *S*_I_(*f* ) shows a general trend characterized by a 1/*f* component at low frequencies and a change from a 1/*f* to a 1/*f* ^3^ dependence at a cut‐off frequency *f*_x_. Therefore, it follows that^25^

(1) $$S_{I}\left(f\right)\;=\;\frac{K}{f^{\gamma }}\frac{1}{1\;+\;{\left(\frac{f}{f_{x}}\right)}^{2}}\;+\;S_{0}$$

where *K* is the temperature‐dependent noise amplitude, γ is an exponent close to unity,^28^ *S*_0_ is the background noise due to the readout electronics, and *f*_x_ is defined as (2*πτ*_eff_)^−1^.^21^

**Figure 2:**
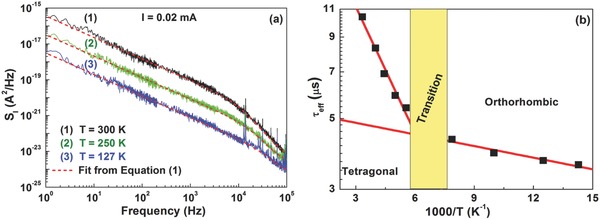
a) Current‐noise spectra of a typical investigated perovskite photovoltaic device at a fixed bias current value (0.02 mA) and at three different temperatures. The best fitting curves (red dashed lines) are obtained with Equation (1). b) Arrhenius plot of the effective lifetime coefficient τ_eff_ in the temperature range between 300 and 70 K. Two distinct behaviors are observed in the room‐temperature tetragonal and in the low‐temperature orthorhombic phases of the perovskite compound.

The good agreement between noise data and the fitting formula of Equation (1) is clearly visible in **Figure** **2**a, where *S*_I_, measured at a fixed bias current of 0.02 mA, is shown for a perovskite solar cell at three different temperatures. The best fitting curves [red dashed lines in Figure 2a] are obtained with values of *f*_x_ varying from (15.3 ± 0.3) kHz at 300 K to (35.1 ± 0.8) kHz at 127 K. This indicates that the effective lifetime decreases by lowering the temperature, from a value of ≈10 µs at room temperature to ≈ 4 µs in the low‐temperature limit. The τ_eff_ temperature dependence is shown in Figure 2b, where two activated behaviors are found as evidenced by the straight lines in the Arrhenius plot. In particular, two distinct activation energies are observed in the low‐temperature orthorhombic [*E*_a_ = (25.5 ± 0.5) meV] and in the room‐temperature tetragonal [*E*_a_ = (53 ± 1) meV] perovskite‐material phases. Similar values of τ_eff_ and of the activation energies have been already reported on CH_3_NH_3_PbI_3_ solar cells by using impedance spectroscopy measurements.^29^ The change from a tetragonal to an orthorhombic structure, for example, has been also observed in literature by X‐ray or neutron diffraction experiments.^30^, ^31^ Recently, the low‐temperature phase transition of perovskite solar cells has been thoroughly investigated by using electric noise spectroscopy. Barone et al. have shown that the dynamics of fluctuations detect the existence of a metastable state in a crossover region between the room‐temperature tetragonal and the low‐temperature orthorhombic phases of the perovskite compound, revealing the presence of a noise peak at this transition.^22^ This finding has been also confirmed by temperature dependent photoluminescence measurements reported in Figure S3 (Supporting Information). Here, the low temperature phase transition is accompanied by an abrupt shift of the photoluminescence from 1.6 eV at 160 K to around 1.7 eV at 140 K. Similar results have been also reported by Milot et al.^32^

Therefore, the behavior of the lifetime τ_eff_ combined with a temperature dependence of the noise amplitude and of the photoluminescence emission gives evidence that a structural phase transition within the perovskite occurs at 160 K. This modification strongly influences the distribution and the types of the defect states within the device. As a consequence, the electric field profile, the charge carrier collection, and the recombination kinetics change. Here, it is important to stress that the perovskite material is characterized by several types of point defects, such as interstitials (*I*_i_), vacancies (*V*_Pb_), cation substitutions (MA_Pb_, Pb_MA_), and antisite substitutions (Pb_I_, I_Pb_).^13^ A fraction of these defects acts as intrinsic acceptor and/or donor shallow defects, thus leading to an uncompensated doping. Another fraction of the defects, characterized by a higher formation energy, can be identified as deep defect states that influence the recombination kinetics and, therefore, the device performances. However, several authors report that films grown under iodine‐rich conditions are prone to a high density of deep electronic traps (recombination centers) while the use of a chloride precursor avoids the formation of specific defects (Pb atom substituted by I) responsible for short diffusion lengths and poor photovoltaic performance.^33^, ^34^ It is worth noting that besides the interstitial Pb_I_ and I_Pb_ point defects, even the fragments of CH_3_NH_3_ and their possible complexes with the iodine atoms can create additional deep traps.^35^

### 2.3 Determination of the Electronic Density of States (DOS) Distribution with Traps

**Figure 3:**
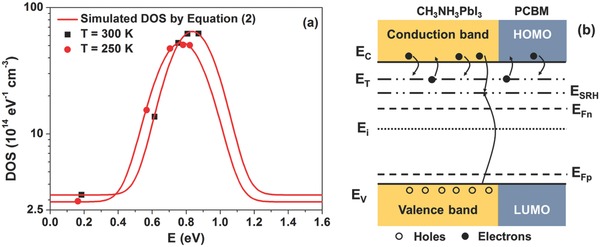
a) Electronic density of states extracted from noise measurements at 300 K (black squares) and at 250 K (red circles), respectively. The best fitting curves, obtained with Equation (2), are also shown as red solid lines. b) Energy‐band diagram of the perovskite‐based device representing the carrier recombination and trapping/detrapping processes.

At zero bias, the contribution of the depletion capacitance *C*_depl_, at the interface between the perovkite layer and the metal contact, is essentially related to the geometric contribution of the PCBM and CH_3_NH_3_PbI_3_ materials.^23^ By increasing the bias voltage, the number of charge carriers accumulated in the active layer increases, thus producing an increase of the chemical capacitance value *C*_μ_, as compared to *C*_depl_. By considering the zero‐temperature approximation of the Fermi function at an occupancy >1%, the chemical capacitance follows the shape of the electronic DOS.^36^ In this respect, it is possible to evaluate the electronic DOS at a given position of the *E*_Fn_ as: $g(E)\;=\;C_{\mu }q^{-2}d^{-1}$, being *q* and *d* the elementary charge and device thickness, respectively, and the capacitance per unit area $C_{\mu }\;=\;\tau_{\mathrm{eff}}\;R_{D}^{-1}$.^37^ In **Figure** **3**a the values of the DOS distribution, derived from noise measurements through τ_eff_, are shown at 300 and 250 K. In disordered semiconductors the DOS is usually modeled with a Gaussian distribution as^1^, ^38^

(2) $$g\left(E\right)\;=\;\frac{N_{0}}{\sqrt{2\pi }\sigma_{n}}\exp\left[-\;\frac{{\left(E\;-\;E_{0}\right)}^{2}}{2\sigma_{n}^{2}}\right]\;+\;g_{0}$$

where *N*_0_ is the electron density per unit volume, *E*_0_ is the center of the DOS, σ_n_ is the disorder parameter, and *g*_0_ represents the DOS background level. The curves resulting from best fitting with Equation (2) are shown in Figure 3a as red solid lines. The best fitting parameters are: *N*_0_ = (1.9 ± 0.1) × 10^15^ cm^−3^, *E*_0_ = (0.84 ± 0.05) eV, and σ_n_ = (124 ± 6) meV for 300 K; *N*_0_ = (1.6 ± 0.1) × 10^15^ cm^−3^, *E*_0_ = (0.77 ± 0.05) eV, and σ_n_ = (120 ± 6) meV for 250 K.

The resulting values of σ_n_ are larger than those reported in literature for the perovskite solar cell with PCBM material as electron transport layer (≈90 meV).^1^ This could be related to the large energy disorder in the device, where the presence of the subgap states^1^ causes non‐negligible recombination losses.^14^ The influence of σ_n_ can be also observed by taking into account the Urbach energy *U*_E_ of the tail states that can be estimated by the quantum efficiency data (shown in Figure S4, Supporting Information). The evaluation of *U*_E_ for the tail states is ≈31 meV with a bandgap *E*_g_ of 1.59 eV for the CH_3_NH_3_PbI_3_ material. This value is larger than what is reported in literature (≈16 meV) and it is a further evidence of the broad distribution of the tail states inside the forbidden gap.^39^ Accordingly to Cody et al., the total disorder can be seen as the sum of two terms, thermal (or dynamic) disorder and static disorder.^40^ The thermal disorder arises from excitations of phonon modes, and the static disorder is due to inherent structural disorder. As already observed by Singh et al. with both absorption and photoluminescence measurements performed on the perovskite material, in the room‐temperature tetragonal phase the main contribution to the disorder can be related to the thermal disorder whereas the static disorder results to be dominant at lower temperatures (*T* < 60 K).^41^ At both the investigated temperatures, the total electron density population *N*_0_ is lower than what found in literature for the best performing CH_3_NH_3_PbI_3_ solar cell (η = 19%), i.e., 10^16^–10^17^ cm^−3^.^1^ This means that the occupation of the highest energy defect states in the DOS tail results to be severely limited by the presence of defects that influence the recombination process. As a consequence, the *N*_0_ value estimated by the noise measurements should be attributed to the density of SRH traps. In literature, several organic and inorganic semiconductors (such as organic polymer, amorphous silicon, and chalcogenide glasses) show a broad distributions of trapping states including deep traps.^42^ In this case, the DOS population results to be dominated by the deep state kinetics.

On the other hand, the value of *g*_0_ has been determined to be ≈3.1 × 10^14^ eV^−1^ cm^−3^ at 300 K and slightly decreases with temperature. As mentioned before, at low bias currents, the main contribution to the capacitance is related to *C*_depl_. Assuming a one‐side abrupt step junction approximation, the depletion zone width is extended within the perovskite material and can be expressed as^43^ $C_{\mathrm{depl}}\;=\;\sqrt{A^{2}q\varepsilon_{0}\varepsilon_{P}\mathrm{N/}2(V_{\mathrm{bi}}\;-\;V_{F})}$, where ε_0_ = 8.85 × 10^−14^ F cm^−2^ is the vacuum permittivity, *A* is the area of the active layer, and ε_P_ = 21.2 is the relative dielectric constant of the CH_3_NH_3_PbI_3_ material.^24^ By considering the value of *V*_bi_, estimated from the *J*–*V* curves under dark conditions (see Figure S1, Supporting Information), the value of the uncompensated doping concentration *N* = (2.1 ± 0.1) × 10^14^ cm^−3^ can be extracted. This is in good agreement with the intrinsic doping concentration (10^9^–10^14^ cm^−3^), reported in literature for the perovskite material, and takes into account the quality of the absorber layer.^10^ In the CH_3_NH_3_PbI_3_ compound a point defects related to Pb or I atom vacancies give the main contribution to the unintentional doping observed in the absorber material that can be either p‐type or n‐type, respectively.^13^, ^44^

It is worth noting that the band tail states, caused by the energy disorder of the PCBM, extend into the bandgap of the perovskite introducing additional electronic states which can participate to the device recombination kinetics. At the interface, the PCBM material results to be homogeneously distributed throughout the film at perovskite grain boundaries and promotes the electron extraction.^45^, ^46^ Therefore, the recombination phenomena take place at the interface perovskite/PCBM and are extended within the CH_3_NH_3_PbI_3_ material. The presence of the exponential tail from the conduction band *E*_C_ can be modeled as shallow defect states *N*_tot_, which act as trapping sites and are located at the energy level *E*_C_ – *E*_T_. Conversely, a small part of *N*_tot_, defined as *N*_SRH_, acts as deep SRH‐type recombination centers.^21^ The SRH defect states are located above the intrinsic Fermi level *E*_i_, at an energy depth *E*_C_ – *E*_SRH_ below the conduction band edge and give the major contribution to the recombination kinetics. In Figure 3b a schematic illustration indicating the electronic transitions between the *E*_T_ and the *E*_SRH_ energy levels with the conduction and valence bands is shown. Under charge carrier injection, the electron quasi‐Fermi level *E*_Fn_ moves across these electronic states modifying their occupation probability. The emission probabilities $e_{n,p}$ and the capture probabilities $c_{n,p}$ for the electron and hole traps influence the density of the filled traps $n_{t}$ and therefore the current fluctuation that can be measured.^21^

### 2.4 Noise Model for the Evaluation of the Defect States

**Figure 4:**
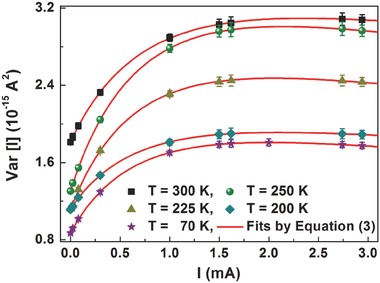
Current dependence of the noise amplitude in the temperature range from 300 to 70 K. The best fitting curves, using Equation (3), are shown as red solid lines. The values for *I* = 0 are the dark noise contributions.

In order to quantify the influence of the electronic transitions between the defect states and the valence and conduction bands, the current‐noise variance Var[*I*] has been computed by integrating *S*_I_ over the whole experimental frequency bandwidth.^21^ The total current (dc bias plus photogenerated) dependence of Var[*I*] is shown in **Figure** **4**, for the same photovoltaic device of Figure 2, at different temperatures ranging from 300 to 70 K. For all the investigated temperatures, an increase of the total current causes a rise of the noise amplitude up to a saturation level Var[*I*]_sat_. This suggests that the charge carrier injection produces a filling of the defect states in the solar cell. The explanation of this behavior can be given in terms of a theoretical model, formulated by combining trapping/detrapping related processes and charge carrier recombination phenomena.^21^ From this interpretation, the data in Figure 4 have been fitted and shown as red solid lines, using the subsequent expression

(3) $$\mathrm{Var}\left[I\right]\;=\;A_{1}\frac{I}{{\left(1\;+\;\frac{I}{I_{0}^{\mathrm{light}}}\right)}^{2}}\;+\;A_{2}\frac{I}{{\left(1\;+\;\frac{I}{I_{A}}\right)}^{2}}$$

In Equation (3), *A*_1_ and *A*_2_ represent the amplitudes of the current fluctuations due to the recombination and trapping/detrapping mechanisms, $I_{0}^{\mathrm{light}}\;=\;\frac{q}{\tau_{\mathrm{eff}}}\mathrm{Ad}\frac{e_{n}}{c_{n}}$ and $I_{A}\;=\;\frac{q}{\tau_{\mathrm{eff}}}\mathrm{Ad}\frac{N}{k}$ with *A* and *d* as the sample area and thickness, respectively.^21^ Assuming that the occupancy of the states is in thermal equilibrium, determined by the Fermi–Dirac distribution, then $e_{n}/c_{n}\;=\;N_{\mathrm{tot}}\;e^{-\frac{E_{C}-E_{\mathrm{SRH}}}{k_{B}\cdot T}}$ is the density of the occupied SRH deep traps *n*_t_, where *N*_tot_ is the effective density of states in the band tail, *k*_B_ is the Boltzmann constant, and *T* is the temperature. The ratio $k\;=\;c_{n}{\mathrm{/c}}_{p}$ is defined as the symmetric ratio and describes the nature of the defect states to be electron or hole attractive trap. As already reported in the case of proton degraded Si‐based photovoltaic devices, from the estimation of the fitting parameters of Equation (3) it is possible to compute the *N*_tot_ and the *N*_SRH_ trap densities as^21^

(4) $$\begin{array}{c} N_{\mathrm{SRH}}=\frac{A_{1}I_{0}^{\mathrm{light}}}{\mathrm{Ad}}{\left(\frac{\tau_{\mathrm{eff}}}{q}\right)}^{2}\;\mathrm{and}\\ N_{\mathrm{tot}}=\frac{A_{2}I_{A}}{\mathrm{Ad}}{\left(\frac{\tau_{\mathrm{eff}}}{q}\right)}^{2} \end{array}$$

**Figure 5:**
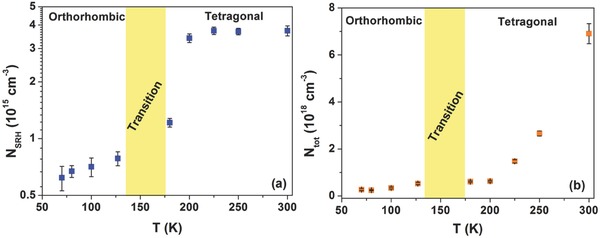
Temperature dependence of a) the recombination trap density *N*_SRH_ and of b) the fluctuating trap density *N*_tot_ in the band tail.

It should be noted that in the temperature region where the phase transition occurs (*T* around 160 K), the experimental measurement of the voltage spectral density, performed at different bias currents, reveals that *S*_V_ is caused by the resistance fluctuation mechanism instead of current fluctuations.^22^ As a consequence, the proposed model, which takes into account the electronic transitions between the defect states and the conduction and valence bands, cannot be applied in the phase transition region. By using Equation (4), the temperature dependence of the trap densities *N*_SRH_ and *N*_tot_ can be extracted and is shown in **Figure** **5**a,b. The ratio *N*_SRH_/*N*_tot_ is less than 1% for the whole investigated temperature range, demonstrating that only a small fraction of the defect states in the band tail acts as recombination centers.

The experimental data show an increase of *N*_SRH_ and *N*_tot_ with increasing temperature. This behavior is consistent with the evidence that the noise amplitude, proportional to the density of the traps within the device, increases with the temperature (see, for example, Figures 2 and 4). At temperatures below 140 K, corresponding to the orthorhombic phase of the perovskite material, the density of defect states *N*_SRH_ seems to be saturated at a value of ≈7 × 10^14^ cm^−3^, as shown in Figure 5a. For *T* > 160 K, after the structural modification of the CH_3_NH_3_PbI_3_ absorber layer, a significant increase of the *N*_SRH_ value is observed. At room temperature *N*_SRH_ is ≃4 × 10^15^ cm^−3^. It is worth noting that the deep defect states, evaluated with the noise modeling, show a value of *N*_SRH_ comparable with the defect states estimated by using the DOS analysis. This finding confirms the assumption that the occupation of the defect states in the band tail is mainly dominated by the deep SRH states and can be attributed to the interstitial point defects (Pb_I_) and the complexes of the CH_3_NH_3_ with the iodine atoms that create relatively deep electron traps.^13^, ^14^, ^35^

As evident in Figure 5b, also the density *N*_tot_ related to the band tail defect states is influenced by the temperature. In particular, below 200 K the *N*_tot_ remains almost constant at ≃3 × 10^17^ cm^−3^ and subsequently begins to increase reaching a value of ≃5 × 10^18^ cm^−3^. This temperature dependence suggests that the trap states are intrinsic to the perovskite and vary with temperature. As also reported by Stranks et al., these defect states may be related to thermally activated atomic vacancies, a common type of point defects in perovskite structures.^13^, ^47^ Moreover, the estimated *N*_tot_ is in good agreement with what found in literature for the devices based on polycrystalline perovskite thin films (10^17^–10^19^ cm^−3^) and obtained with alternative experimental techniques, such as temperature dependent admittance spectroscopy and time‐resolved photoluminescence decay measurements.^10^, ^18^ The corresponding value of the energy depth of the traps below the conduction band can be estimated as $E_{C}\;-\;E_{\mathrm{SRH}}\;=\;k_{B}\mathrm{Tln}(N_{\mathrm{tot}}{\mathrm{/n}}_{t})$ = (230 ± 6) meV at 300 K with a trap density of *N*_SRH_ ≈ 4 × 10^15^ cm^−3^. This finding is consistent with the results reported in literature for polycrystalline perovskite thin films, which are characterized by several peaks in the energy distribution located at 167, 240, and 660 meV below the conduction band edge.^10^ Here, it is important to underline that the corresponding value of the symmetric ratio *k* for the *N*_SRH_ defect states varies between 2 and 10 and decreases as the temperature increases. This represents a further indication that the deep states act as electron‐attractive traps.^14^, ^48^

### 2.5 Energy Distribution of the Defect States

**Figure 6:**
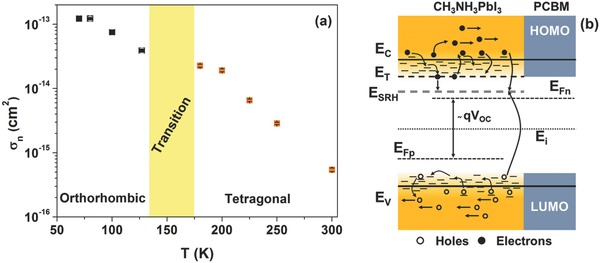
a) Temperature dependence of the electron capture cross‐section σ_n_. b) Energy‐band diagram of the perovskite‐based device representing the electronic transitions between the traps and the conduction and valence bands in a two stage model for deep states.

As observed by the noise measurements, shown in Figure 4, the current fluctuations in the perovskite solar cell are strictly related to the trap‐assisted recombination processes and modeled by two energy levels, which act as trapping (*E*_T_) and recombination (*E*_SRH_) centers. Under low charge carrier injection, it can be assumed that $\tau_{\mathrm{eff}}\;=\;\tau_{n}\;(1\;+\;I_{0}{\mathrm{/I}}_{A})$, with $\tau_{n}\;=\;{\left(c_{n}N_{\mathrm{tot}}\right)}^{-1}$.^21^ Here *c*_n_ is defined as the product of the electron capture cross‐section σ_n_ and its thermal velocity *v*_th_, that varies in temperature as *T* ^1/2^. Therefore, the σ_n_ values can be estimated once the temperature dependence of $N_{\mathrm{tot}}$ has been considered. In **Figure** **6**a σ_n_ versus *T* is shown. Several studies report the decrease of σ_n_ with the increase of the temperature as for example the case of the hole trap in GaP material.^49^, ^50^ This decay behavior suggests that the electron capture in the defect states is phonon‐assisted demonstrating that the phonon scattering influences the charge carrier transport in the CH_3_NH_3_PbI_3_‐based solar cells. Recently, this strong electron–phonon interaction has been observed by using photoluminescence and far‐infrared spectroscopy measurements.^15^, ^51^

In the perovskite solar cell, the CH_3_NH_3_PbI_3_ absorber layer can be considered as hybrid organic–inorganic material characterized by a large value of the energy disorder. As reported in literature, the transport mechanism occurs by multiple trapping process and hopping transport through defect sites (distributed in space and energy).^42^, ^52^ In Figure 6b the trapping/detrapping and recombination mechanisms between shallow and deep defect states under charge carrier injection are shown. In this respect, Gibb et al. proposed a model which describes the interactions between the charge carriers and the phonons in presence of distributed defect states.^50^ Here, the capture of the charge carrier occurs in two stages in which the electron is first captured by a shallow defect (localized at *E*_C_ – *E*_T_) and then it can be emitted in the band tail state or thermalize to a deeper ground state (*E*_C_ – *E*_SRH_) through a multiphonon emission process.^50^ As a consequence, σ_n_ can be expressed as^50^

(5) $$\sigma_{n}\;=\;\frac{v}{v_{\mathrm{th}}N_{c}}\;e^{\left(\frac{E_{C}-E_{T}}{k_{B}T}\right)}\;+\;\sigma_{c}$$

where ν is the capture rate (assumed independent on *T*) into a deep level from the shallow center and *N*_c_ is the effective density of states in the conduction band which varies as *T* ^3/2^. By taking into account the temperature dependence of *v*_th_ and *N*_c_ in Equation (5), it follows that $\sigma_{n}\;\approx \;T^{-2}\exp[(E_{C}\;-\;E_{T})/k_{B}T]$ at high temperatures. On the other hand, by decreasing the temperature σ_n_ becomes approximately equal to σ_c_. This means that shallow level capture is inevitably followed by a capture into the deep defect states. This process leads to a loss of the energy of the carrier with the emission of phonons during the thermalization.^48^

**Figure 7:**
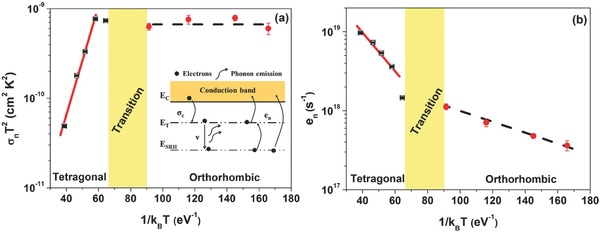
Arrhenius plot of a) σ_n_ *T* ^2^ for the shallow defect states at 140 meV and of b) the *e*_n_ for the deep defect states at 220 meV below the conduction band edge. Two distinct behaviors are observed in the room‐temperature tetragonal and in the low‐temperature orthorhombic phases of the perovskite compound. The inset shows the electron capture and emission mechanisms from the conduction band caused by the shallow and deep defect states with the multiphonon emission process.

As evidenced in **Figure** **7** the dynamics of the defect states involved in the trapping mechanisms is also related to the structural phase of the absorber material. In Figure 7a, the Arrhenius plot of σ_n_ *T* ^2^ for the perovskite solar cell is shown, revealing a clear change of the slope when a structural modification occurs. In particular, at low temperature corresponding to the tetragonal phase of the CH_3_NH_3_PbI_3_ material, the quantity σ_n_ *T* ^2^ is characterized by a constant dependence of type σ_c_ = σ_0_ *T* ^−^ *^α^* with α = 2. This implies that the capture process of the charge carriers is a cascade capture mode characterized by a strong phonon interaction.^50^ Moreover, at higher temperatures, where the absorber layer shows an orthorhombic phase, the exponential contribution in Equation (5) becomes dominant, leading to an estimation of the energy depth of the shallow defects as *E*_C_ – *E*_T_ = (141 ± 4) meV.

The probability that an electron can be emitted into the conduction band, once it is captured by the shallow defect, decreases with the gradual diffusion down of the trapped carrier toward deeper states. The emission probability from the deep states can be expressed as $e_{n}\;=\;N_{c}c_{n}\exp[-(E_{C}\;-\;E_{\mathrm{SRH}})/k_{B}T]$, with $c_{n}\;=\;v_{\mathrm{th}}\;\sigma_{n}$. By using Equation (5), at higher temperatures the emission rate is estimated as $e_{n}\approx \;\exp[-(E_{\mathrm{SRH}}\;-\;E_{T})/k_{B}T]$. Conversely, in the lower temperature range, where only the shallow defects are involved in the trapping and recombination mechanisms, the temperature behavior of *e*_n_ is estimated as $e_{n}\approx \exp[-(E_{C}-E\prime )/k_{B}T]$, where *E*′ is defined to be within a distance of few *k*_B_ *T* (the characteristic thermal energy) of the conduction band. In Figure 7b the Arrhenius plot of *e*_n_ from 70 to 300 K is shown. For *T* > 160 K, deeper defect states located at about 74 meV below the energy depth of the shallow centers are observed. This means that the active SRH‐type recombination centers lie at ≈220 meV below the conduction band edge, consistent with the estimations reported before. Conversely, when the structure of the absorber material changes to the orthorhombic phase, the dominant recombination kinetics can be ascribed to shallow defects located at ≈15 meV below the band edge, having a defect density of ≈7 × 10^14^ cm^−3^. The observed cascade capture process indicates that the trapping of the charge carriers is phonon assisted directly followed by their recombination.

A further increase of the temperature causes a phase modification of the CH_3_NH_3_PbI_3_ absorber layer with a consequent formation of deep states that are strictly connected to shallow states. In the latter case, the recombination of the charge carriers due to the deep states is preceded by an electron trapping in shallow defects and subsequent thermalization. Usually the shallow defects do not participate to the recombination processes and contribute to the long diffusion length and high open‐circuit voltage of the perovskite solar cells.^4^ However, their dynamics combined with the electron–phonon interactions in presence of the deeper states leads to an increase of the nonradiative recombination pathways with a negative influence on the solar cell performance.

### 2.6 Implications to Power Conversion Efficiency

**Figure 8:**
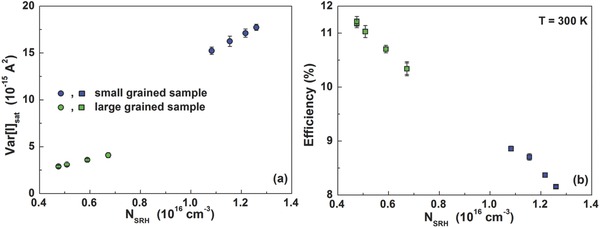
a) Experimental value of the saturated current‐noise level Var[*I*]_sat_ as a function of the SRH defect density *N*_SRH_ (colored circles). b) Correlation between the room‐temperature power conversion efficiency and the *N*_SRH_ defect density (colored squares). Blue symbols refer to the small grained sample (*d*_grain_ ≈ 150 nm), while green symbols refer to the large grained sample (*d*_grain_ ≈ 370 nm).

Solar cell performance depends strongly on the recombination kinetics and the present defect states. To investigate this, two distinct perovskite solar cells have been fabricated. The first sample, deposited from a 1.1 m precursor solution shows an average grain diameter *d*_grain_ of 370 nm and a thickness of 430 nm. The second sample, deposited from a 0.8 m precursor solution shows an average grain diameter of 150 nm and a thickness of around 210 nm. The grain diameter distribution is shown in Figure S5 (Supporting Information). It should be noted that the grain size distribution widened in conjunction with diameter rise (see Figure S6, Supporting Information). In the following, the samples will be referred as large and small grained. PEDOT:PSS, PCBM, and BCP layers have been processed identically for both samples. **Figure** **8**a depicts the saturated current‐noise level Var[*I*]_sat_ as a function of the SRH trap density *N*_SRH_, estimated with the model of Equation (4), for the large and the small grained sample. A clear correlation between the noise amplitude and the *N*_SRH_ defect density can be observed. Samples based on smaller CH_3_NH_3_PbI_3_ grains are characterized by a greater value of the Var[*I*]_sat_ compared to the solar cells with large CH_3_NH_3_PbI_3_ grains. Additionally, the defect density *N*_SRH_ decreases with an increase of the grain diameter suggesting that the recombination losses can be related to the defects/impurities in the crystal bulk and/or along the grain boundaries of the perovskite material. This finding has been also confirmed by the analysis of the charge carrier diffusion length in perovskite material by using surface photovoltage measurements, where an increased diffusion length for the larger grain size sample has been reported.^53^ As a further evidence of this correlation, an increase of the *U*_E_ value, estimated by the quantum efficiency data shown in Figure S4 (Supporting Information), with decreasing of the grain size of the absorber layer has been observed. This finding is consistent with the results reported in literature, where an increase of the electron–hole diffusion length, longer lifetime, and smaller *N*_SRH_ density is attributed to the growth of grain size of the perovskite material from polycrystalline to single crystal thin films.^22^, ^54^, ^55^ Consistent with the smaller *N*_SRH_ density reported for the large grained sample, an increased *V*_OC_ value of 0.89 V compared to 0.68 V has been observed. This finding can be seen in Figure S7 (Supporting Information). The recombination kinetics within the device therefore limits the occupation of the highest energy defect states in the DOS tail. Under photogenerated charge carriers, the sub‐bandgap states act as a deep SRH‐type recombination centers and cause a charge carrier loss which severely reduces the quasi‐Fermi level splitting within the device, and hence the open circuit voltage of the solar cells.^1^ In Figure 8b the power conversion efficiency values as a function of the *N*_SRH_ defect density for both the solar cells investigated are shown. As can be noted, devices with a large grained absorber layer show higher value of η and, therefore, lower recombination losses through *N*_SRH_ centers compared to the device with thinner absorber layer and smaller grain sizes.^56^ The results are listed in Table S1 (Supporting Information).

## 3 Conclusion

A detailed temperature characterization of defect states and their energy distribution in the organic–inorganic hybrid perovskite solar cells have been performed under dark conditions and under light illumination by means of fluctuation spectroscopy. Under charge carrier injection, the temperature dependence of the carrier lifetime, extracted by the noise spectra, evidences the structural phase transition of the perovskite material at 160 K between the orthorhombic and the tetragonal phases, thus validating the low frequency noise spectroscopy as an effective and nondestructive tool.

The measured electronic density of states distribution evidences a large energy disorder in the device, where the presence of the sub‐bandgap states causes non‐negligible recombination losses. The observed temperature behavior of the capture cross‐section suggests that the electron capture in the perovskite material is phonon assisted. At low temperatures, corresponding to the orthorhombic phase structure of the absorber layer, the trapping process can be ascribed to shallow defects and the recombination mechanism follows the cascade capture mode characterized by a strong electron–phonon interaction. Conversely at higher temperatures, the capture of the charge carrier occur in two steps in which the electron is first captured by a shallow defect and subsequently it can be either emitted or thermalize down to a deeper band tail state with the phonons emission and recombines subsequently. Given the temperature dependence of the electron capture cross‐section and the peculiar Arrhenius plots, the proposed two‐stage model results to be the only one consistent among the phonon‐assisted capture models. Moreover a clear correlation between the morphological structure of the CH_3_NH_3_PbI_3_ grains, the energy disorder of defect states, and the device parameters in the perovskite solar cell has been demonstrated.

## 4 Experimental Section

*Device Fabrication*: Perovskite solar cells were prepared on patterned glass/ITO substrates. First, a 60 nm thick PEDOT:PSS layer (Heraeus AI 4083) was prepared by spin coating at 3000 rpm. The obtained films were annealed at 150 °C for 20 min and then transferred to inert atmosphere for further processing. There, polycrystalline thin films of CH_3_NH_3_PbI_3_ were deposited from a 0.8 m (small grained sample) and a 1.1 m (large grained sample) precursor solution. The stoichiometric precursor solutions contained PbI_2_ (Sigma‐Aldrich, 99.999%) and CH_3_NH_3_I (synthesized from CH_2_NH_2_ and HI) in γ‐butryolactone and dimethyl sulfoxide with a volume ratio of 70/30. Spin coating was performed at 1000 rpm for 10 s, 2000 rpm for 20 s, and 5000 rpm for 20 s. In the last spin‐coating stage 150 µL of chlorobenzene were dripped on top of the sample.^57^ After crystallization of CH_3_NH_3_PbI_3_ at 100 °C for 10 min the electron selective contacts were deposited. PC_61_BM (Lumtec, 99.5%, 20 mg mL^−1^ in chlorobenzene) was spin coated at 2500 rpm. After subsequent annealing at 100 °C a thin layer of BCP (Sigma‐Aldrich, 0.5 mg mL^−1^ in ethanol) was deposited by spin coating at 4000 rpm. Finally 100 nm of Ag was thermally evaporated through a shadow mask at a base pressure below 10^−6^ mbar. The active area was confined to 0.16 cm^−2^ by the overlapping metal and ITO contacts.

*Device Characterization*: The perovskite solar cells were characterized under a simulated solar spectrum (AM1.5G, Newport LCS‐100 class ABB sun simulator). The light intensity hereby was calibrated using an Si photodiode from Fraunhofer Institute for Solar Energy Systems ISE CalLab Photovoltaic Cells. Current voltage scans were acquired in forward and reverse directions using a voltage sweep of 85 mV s^−1^. A calibrated “ORIEL QEPVSI‐b” setup was used to acquire the External Quantum Efficiency (EQE) spectra. Measurements were performed without bias illumination at short circuit.

To prevent degradation by oxygen and moisture, EQE and *J*–*V* measurements were performed under inert atmosphere. For noise measurements, the samples were encapsulated using a cover glass and a two‐component epoxy glue. A closed‐cycle refrigerator was used to vary the temperature, whose stabilization was realized by a resistance heater controlled in a closed feedback loop (1 K of achieved stability). The experimental setup was made by a low‐noise Keithley dc current source for the biasing of the samples, by a digital multimeter for the dc voltage drop recording, by a low‐noise PAR5113 Preamplifier and a dynamic signal analyzer HP35670A for the analysis of the ac voltage signal. A commercial cool white light emitting diode was used as light source for low‐temperature investigations. The spurious electrical noise generated by the contacts was minimized by resorting to a specific procedure, based on a sequence of two‐ and four‐probe measurements. Reliability of this technique has already been tested in detail on several materials and devices.^58^ Scanning electron images were recorded with an Hitachi S‐4100 at 5 kV acceleration voltage. In case of top view images the PCBM and BCP layers were removed prior to analysis. Evaluation of the grain diameter distribution was obtained by processing top view Scanning Electron Microscopy (SEM) images with ImageJ.

## Conflict of Interest

The authors declare no conflict of interest.

## Supporting information

Supplementary
