# Supplementary material for: Correlation between Electronic Defect States Distribution and Device Performance of Perovskite Solar Cells
Source: Adv Sci (Weinh). 2017 Jul 6;4(10):1700183. doi: 10.1002/advs.201700183 (PMC5644241; doi:10.1002/advs.201700183)
Supplement: Supplementary file 1 — Supplementary [file ADVS-4-na-s001.pdf]

Copyright WILEY-VCH Verlag GmbH & Co. KGaA, 69469 Weinheim, Germany, 2017.

ADVANCED SCIENCE Open Access

Supporting Information

for *Adv. Sci.,* DOI: 10.1002/advs.201700183

Correlation between Electronic Defect States Distribution and Device Performance of Perovskite Solar Cells

Giovanni Landi, Heinz Christoph Neitzert, Carlo Barone, Costantino Mauro, Felix Lang, Steve Albrecht, Bernd Rech, and Sergio Pagano*

Copyright WILEY-VCH Verlag GmbH & Co. KGaA, 69469 Weinheim, Germany, 2016.

Supporting Information

Correlation between electronic defect states distribution and device performance of perovskite solar cells

Giovanni Landi, Heinz Christoph Neitzert, Carlo Barone, Costantino Mauro, Felix Lang, Steve Albrecht, Bernd Rech, Sergio Pagano*

Dr. G. Landi, Prof. Dr. H. C. Neitzert
Dipartimento di Ingegneria Industriale (DIIn), Università di Salerno, Via Giovanni Paolo II 132, 84084 Fisciano (SA), Italy
E-mail: glandi@unisa.it

Dr. C. Barone, C. Mauro, Prof. Dr. S. Pagano
Dipartimento di Fisica "E.R. Caianiello" and CNR-SPIN Salerno, Università di Salerno, Via Giovanni Paolo II 132, 84084 Fisciano (SA), Italy

F. Lang, Dr. S. Albrecht, Prof. Dr. B. Rech
Helmholtz-Zentrum Berlin für Materialien und Energie GmbH, Institut für Silizium Photovoltaik, Kekuléstr. 5, 12489 Berlin, Germany.

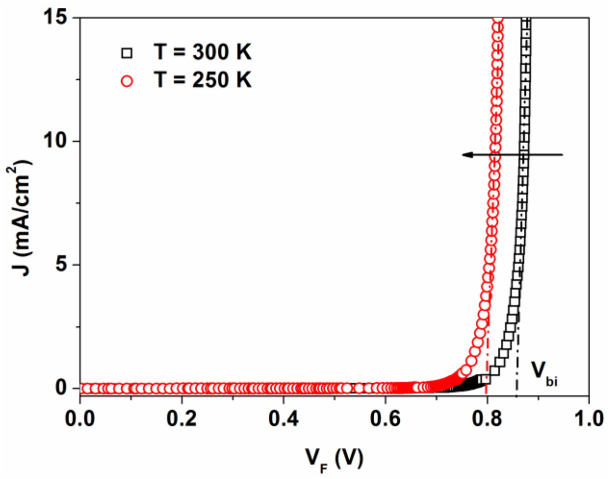

Figure S1. Current density-voltage characteristics of the measured perovskite solar cell at 300 and 250 K. V_{bi} and $V_F = V - R_sJ$ correspond to the built-in voltage and the forward bias voltage, respectively.

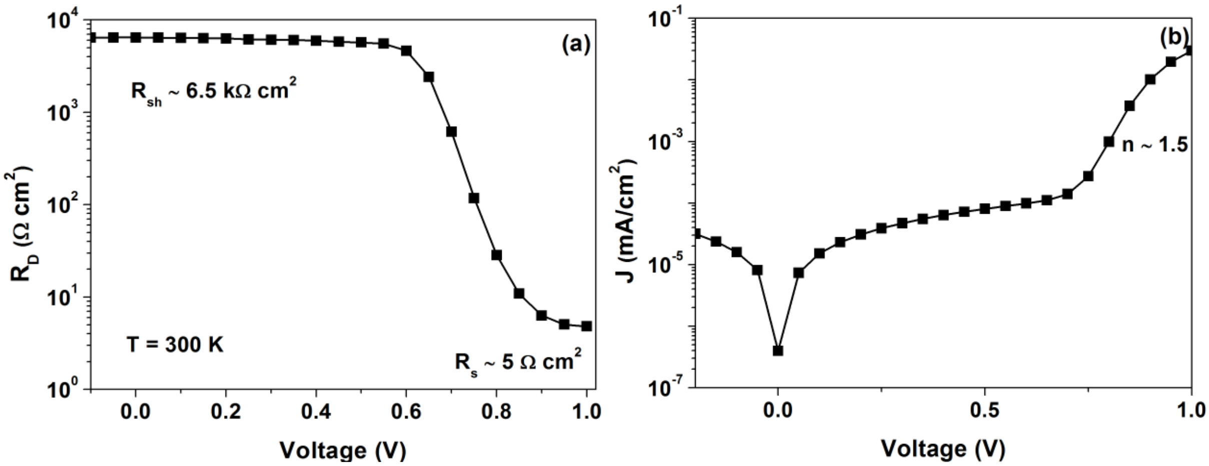

Figure S2. (a) Differential resistance as a function of the forward bias voltage and (b) semilogarithmic plot of the current density-voltage characteristics of the measured perovskite solar cell under dark conditions at 300 K. R_s, R_{sh}, and n correspond to the series resistance, shunt resistance, and diode ideality factor.

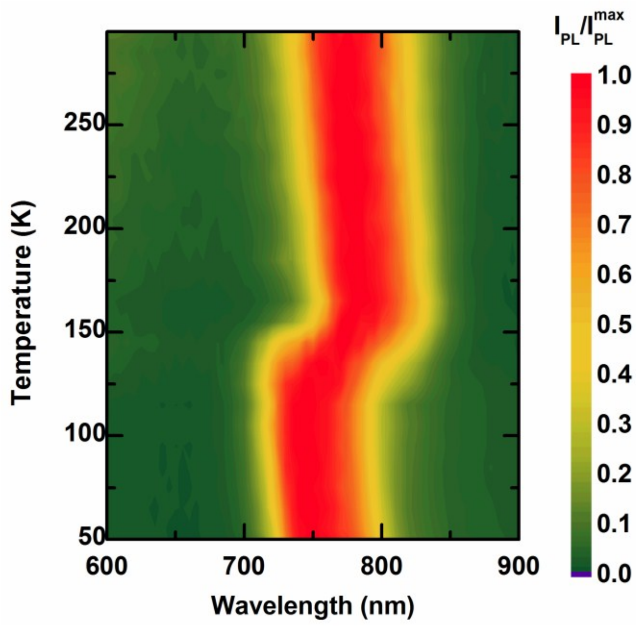

Figure S3. Normalized temperature dependent photoluminescence of $CH_3NH_3PbI_3$. PL spectra were recorded after pulsed excitation with 504 nm using a suitable dye. The pulse length was 0.5 ns, and the energy was reduced to around 14 µJ per pulse to avoid degradation. The temperature was controlled using a liquid helium controlled finger cryostat from Oxford Instruments.

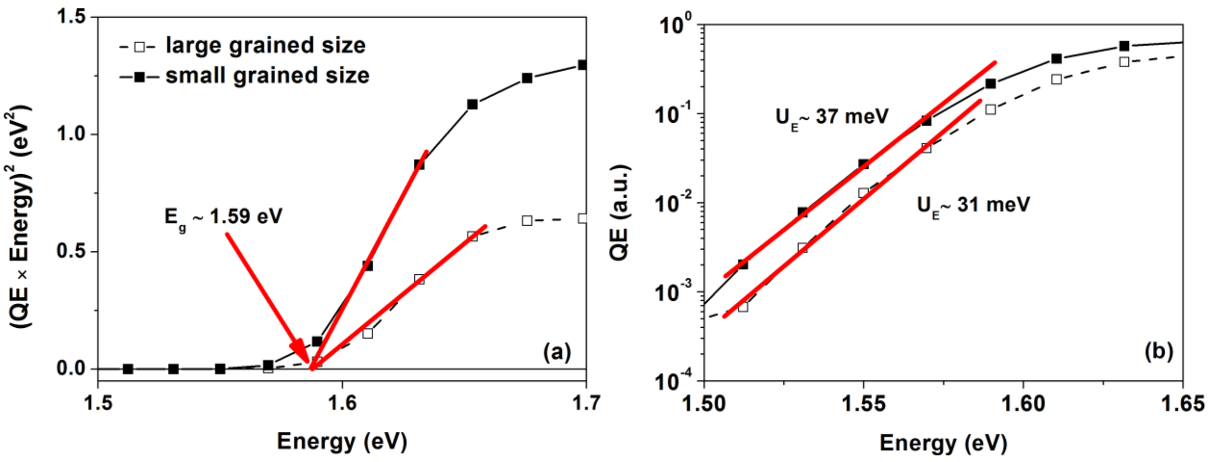

Figure S4. (a) $(QE \times E)^2$ as a function of the photon energy E data for both the investigated perovskite solar cells. The intercept on the energy axis indicates a bandgap of 1.59 eV for both the large (d_{grain} ~ 370 nm) and small (d_{grain} ~ 150 nm) grained $CH_3NH_3PbI_3$ absorber material. (b) Quantum efficiency showing an Urbach energy U_E of 31 meV and 37 meV for the large and small grained absorber layer, respectively.

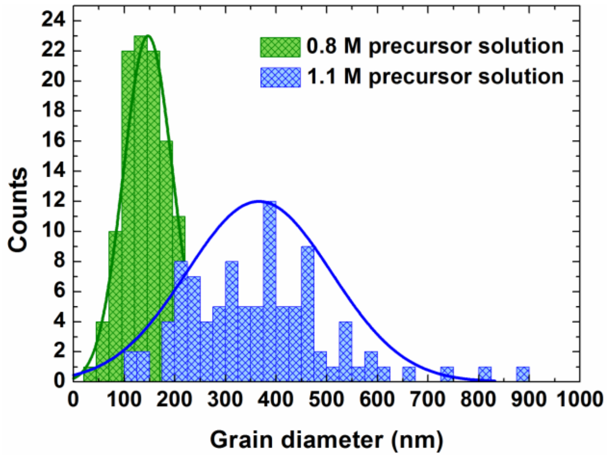

Figure S5. Histogram of the grain diameter distribution for samples deposited from an 1.1 M (blue) and 0.8 M (green) precursor solution. Solid lines show optimized normal distribution.

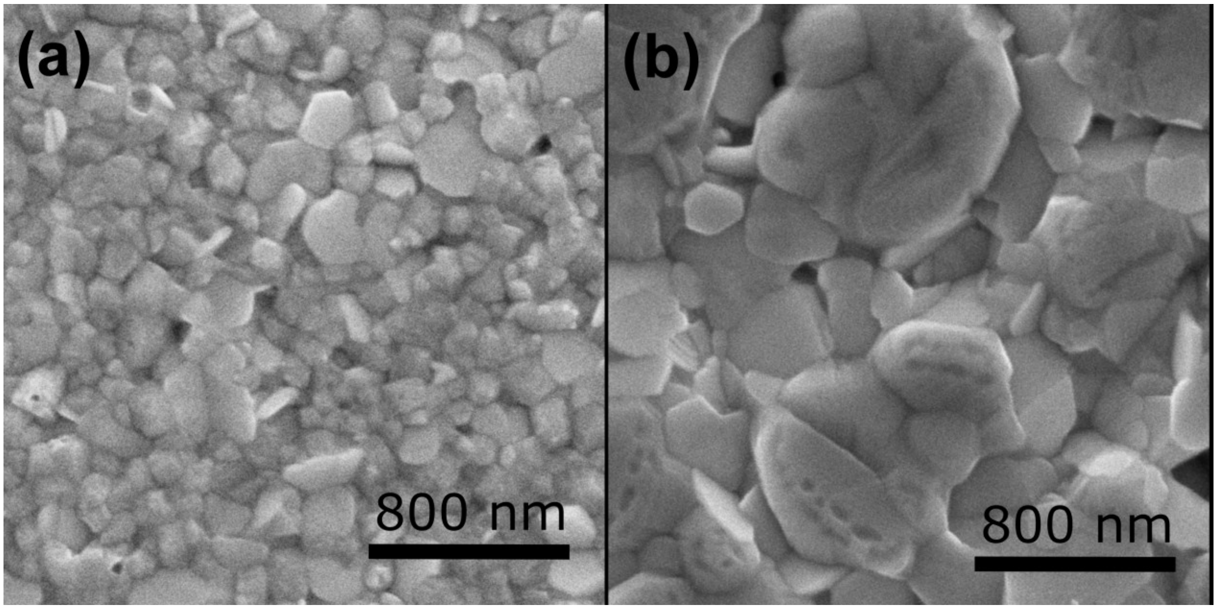

Figure S6. Top view scanning electron micrographs of (a) small (d_{grain} ~ 150 nm) and (b) large (d_{grain} ~ 370 nm) grained $CH_3NH_3PbI_3$ absorber material.

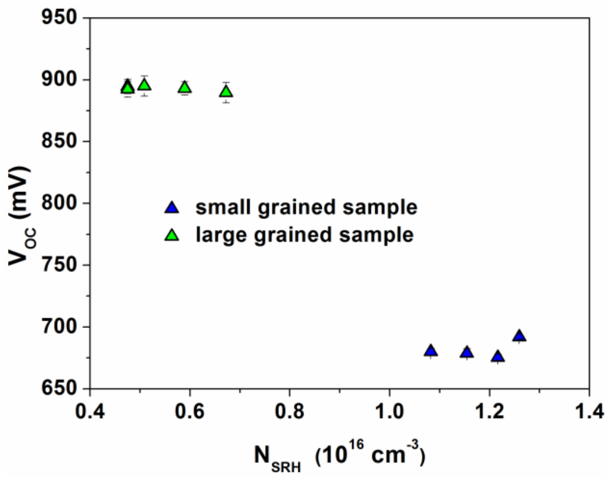

Figure S7. Correlation between the room-temperature open circuit voltage and the N_{SRH} defect density. Blue symbols refer to the small grained sample (d_{grain} ~ 150 nm), while green symbols refer to the large grained sample (d_{grain} ~ 370 nm).

Table S1. Correlation between the morphological structure of the perovskite grains, the defect states, and the solar cell performance measured at 300 K.

Sample type	d_{grain} (nm)	Thickness (nm)	U_E (meV)	$Var[I]_{sat}$ (× 10^{-15} A^{-2})	N_{SRH} (× 10^{16} cm^{-3})	τ_{eff} (μs)	V_{OC} (mV)	η (%)
Large grained	370	430	31	3.6	0.6	10.4	890	10.7
Small grained	150	210	37	17	1.2	6.5	680	8.4
